# Improvement of neutrophil gelatinase-associated lipocalin sensitivity and specificity by two plasma measurements in predicting acute kidney injury after cardiac surgery

**DOI:** 10.11613/BM.2018.030701

**Published:** 2018-10-15

**Authors:** Giovanni Introcaso, Matteo Nafi, Alice Bonomi, Camilla L’Acqua, Luca Salvi, Roberto Ceriani, Davide Carcione, Annalisa Cattaneo, Maria Teresa Sandri

**Affiliations:** 1 Unit of Laboratory Medicine, Centro Cardiologico ‘’Monzino’’, IRCCS, Milan, Italy; 2 Intensive Care Unit, Centro Cardiologico ‘’Monzino’’, IRCCS, Milan, Italy; 3 Units of Biostatistics, Centro Cardiologico ‘’Monzino’’, IRCCS, Milan, Italy

**Keywords:** neutrophil gelatinase-associated lipocalin, acute kidney injury, cardiovascular surgery, diagnostic accuracy, AKI biomarkers

## Abstract

**Introduction:**

Acute kidney injury (AKI) remains among the most severe complication after cardiac surgery. The aim of this study was to evaluate the neutrophil gelatinase-associated lipocalin (NGAL) as possible biomarker for the prediction of AKI in an adult cardiac population.

**Materials and methods:**

Sixty-nine consecutive patients who underwent cardiac surgeries in our hospital were prospectively evaluated. In the intensive care unit (ICU) NGAL was measured as a new biomarker of AKI besides serum creatinine (sCrea). Patients with at least two factors of AKI risk were selected and samples collected before the intervention and soon after the patient’s arrival in ICU. As reference standard, sCrea measurements and urine outputs were evaluated to define the clinical AKI. A Triage Meter for plasma NGAL fluorescence immunoassay was used.

**Results:**

Acute kidney injury occurred in 24 of the 69 patients (35%). Analysis of post-operative NGAL values demonstrated an AUC of 0.71, 95% CI (0.60 - 0.82) with a cut-off = 154 ng/mL (sensitivity = 76%, specificity = 59%). Moreover, NGAL after surgery had a good correlation with the AKI stage severity (P ≤ 0.001). Better diagnostic results were obtained with two consecutive tests: sensitivity 86% with a negative predictive value (NPV) of 87%. At 10-18 h after surgery sCrea measurement, as confirmatory test, allowed to reach a more sensitivity and specificity with a NPV of 96%.

**Conclusions:**

The assay results showed an improvement of NGAL diagnostic accuracy evaluating two tests. Consequently, NGAL may be useful for a timely treatment or for the AKI rule out in ICU patients.

## Introduction

Acute kidney injury (AKI) is worldwide among the most frequent and serious postoperative complications in surgical patients (*1*, *2*). Acute kidney injury describes a sudden pathological process detected functionally by the rise in serum creatinine (sCrea) and decline in the estimated glomerular filtration rate (eGFR) and in the urine output. It is associated with adverse outcomes in patients undergoing cardiovascular/cardiothoracic surgery. However, increases in serum creatinine following renal injury are delayed and not efficacy, preventing from a timely and appropriate early diagnosis and treatment (*3*). The need for more sensitive biomarkers in the early post-operative period and for diagnostic protocol to monitor renal function has prompted several researchers to look for early and reliable markers of postoperative kidney dysfunction. In the last decade, neutrophil gelatinase-associated lipocalin (NGAL) protein has emerged as a promising biomarker (*4*-*6*). Basic research has shown that the morphologic and functional response of renal tubular cells to the renal ischemia depends on the intensity and severity of ischemia and includes loss of cell polarity, cell death, proliferation, differentiation (*7*-*9*). The rationale for the clinical application of NGAL in the diagnostic and/or prognostic evaluation of AKI is due to an intensive gene up-regulation following to injurious events that determine a high secretion of this protein in the damaged tissue (*7*, *8*). Despite several studies have indicated the utility of NGAL measurement in the early detection of AKI, to date, clear consensus on clinical applications of NGAL determination does not exist (*10*-*13*). Particularly critical is the identification among subject who may already present an increased risk of postoperative complications, due to their clinical status or to underlying disease, those who actually will develop postoperative AKI. The aim of this study was to investigate the usefulness as diagnostic value of plasma NGAL in the early prediction of AKI in adults undergoing cardiac surgery at high risk of AKI development, in association with the conventional marker serum creatinine. The study had been conducted applying to biochemical test NGAL and sCrea the basic measures of diagnostic accuracy.

## Materials and methods

Between January 2014 and September 2015, 92 consecutive patients undergoing cardiac surgery, at the Centro Cardiologico Monzino, and matching the inclusion criteria were prospectively enrolled in the study. Among them 23 had missing results or clinical data and were excluded from the analysis. This study was approved by the local Ethic Committee and all patients provided written informed consent. Patients presenting two or more of the following criteria were included: 1) age > 70 years, 2) eGFR < 60 mL/min/1.73m^2^ (estimated by Modification of Diet in Renal Disease, MDRD formula), 3) ejection fraction (EF) < 41%, 4) redo operation, 5) combined surgery. In the surgical program each patient was classified according to the European System for Cardiac Operative Risk Evaluation (Euroscore). This risk model predicts the mortality in cardiac surgery.

The diagnosis of AKI was made according to the Kidney Disease-Improving Global Outcomes (KDIGO) criteria (*14*). The present international guideline based on serum creatinine and urine output, data available in the Hospital database, represents the gold standard for AKI diagnosis. The AKI definition was between 1 to 3 according to 24h sCrea modifications and urine output (*14*). Acute kidney injury equal 0 means modifications of serum creatinine and urine output lower than AKI 1. When AKI was 0 the patient was allocated in the group No-AKI, otherwise when AKI was ≥ 1 the patient was allocated in the group AKI. Serum creatinine modifications of 1, 2, 3 times baseline and progressive urine output reduction identified the AKI degree with stage: 1 or 2 or 3. Acute kidney injury stage of 2 and 3 identified patients with high severity of kidney injury.

Plasma NGAL was evaluated in blood samples taken at two time points: before surgery at general anaesthesia induction (baseline or pre-NGAL) and within 4h from the arrival of the patients in ICU (post-NGAL). Blood samples collected in 3 mL tube (BD Vacutainer, K_3_EDTA 5.4 mg, as anticoagulant, Becton Dickinson and Company, Franklin Lakes, USA) were centrifuged at 1000×g at room temperature for 10-15 minutes, aliquoted in 0.5 mL tubes and stored at - 80 °C until assayed. Neutrophil gelatinase-associated lipocalin was measured using a point-of-care test method (Triage Meter NGAL Test, Biosite, Alere Health, San Diego, USA), a rapid fluorescence based immunoassay.

Serum creatinine concentrations were measured before surgery, after 10-18 hours and every day for the three consecutive postoperative days, on Unicell DxC clinical system (Beckman Coulter, Indianapolis, USA) using a colorimetric Jaffe method with isotope dilution mass spectrometry standardization. A tube of 4.5 mL lithium heparin with separator gel was used from the laboratory routine (Becton Dickinson and Company, Franklin Lakes, USA). Quality control charts with two levels are assessed daily by the use of random and systematic control rules and critical evaluation monthly, using the Synchron control kit (Beckman Coulter, Indianapolis, USA). The sCrea analytical variability (CV%) applied for the imprecision profile at different plasma concentrations ranged from 1.1% to 3.6%.

## Statistical analysis

Continuous variables are presented as mean ± SD, and were compared using the t-test for independent samples. Variables not normally distributed are presented as median and interquartile ranges, and compared with the Wilcoxon rank-sum test. Categorical data were compared using the chi-square test or the Fisher exact test, as appropriate. Correlations between variables were determined using the Pearson test or Spearman’s rank test, as appropriate. Receiver operating characteristic (ROC) curves were calculated and the area under curve (AUC) with 95% CI was used to measure the ability of NGAL to predict AKI.

The cut-off of NGAL was detected by Euclidean distance, able to balance the sensitivity and specificity of predicting a case. Tests were two-sided. P-values < 0.05 were considered statistically significant. Analyses were performed by SAS version 9.4 (SAS Institute Inc., Cary, NC). A critical NGAL interpretation, based on two consecutive tests and according to expert opinion, was made, considering clinically significant an increase in NGAL (positive test) if the second value (post operation) was ≥ 100 ng/mL (*15*).

## Results

**Figure 1:**
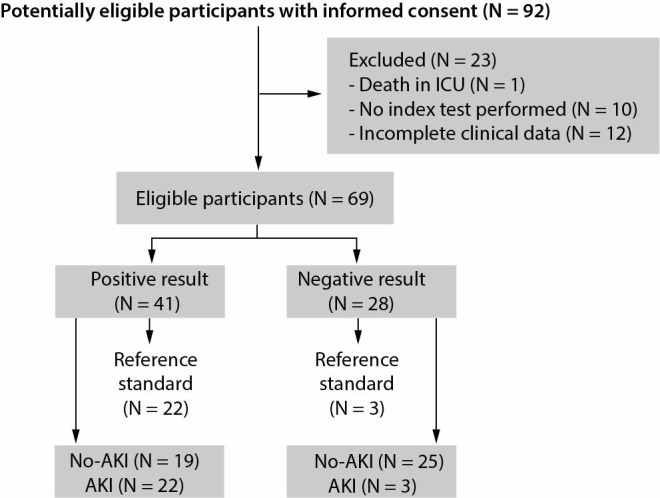
The flow diagram shows the neutrophil gelatinase-associated lipocalin (NGAL) evaluation (index test) performing two consecutive measurements. The positive result was obtained through the evaluation of ΔNGAL as biomarker increase. AKI - acute kidney injury. No-AKI - no acute kidney injury. ICU - intensive care unit.

To illustrate the study steps, we had built a flow diagram of the participants, showing the number of NGAL tests performed as index test and the number of AKI diagnosis using the sCrea and urine output modifications as reference standard (Figure 1). The clinical and main characteristics of the study population are shown in Table 1. No significant differences were found regarding age, weight, Euroscore, baseline concentrations of sCrea and NGAL. No significant differences occurred for surgical procedures performed among the patients in AKI and No-AKI groups. Surgical procedures were as follows: 19 (0.28) patients underwent aortic valve replacement, 11 (0.16) patients underwent transcatheter aortic valve implantation (TAVI), 19 (0.28) patients underwent cardiac artery bypass graft, 10 (0.14) patients underwent mitral valve replacement or valvuloplasty, 2 (0.03) patients underwent aortic ascendant replacement, 2 (0.03) patients underwent pericardiectomy and 6 (0.08) patients underwent balloon valvuloplasty with or without cardiopulmonary bypass (CPB).

**Table 1:** Characteristics of the study participants

	**AKI**	**No-AKI**	**P**
N	24	45	/
Gender, male (proportion)	15 (0.62)	29 (0.64)	/
Age, years	76 (55 - 81)	78 (58 - 89)	0.056
Weight, kg	75 (61 - 80)	69 (63 - 78)	0.317
Euroscore	5 (3 - 7)	6 (3 - 9)	0.703
NGAL_baseline_, ng/mL	94 (72 - 179)	92.5 (73.5 - 165)	0.659
sCrea (CKD), µmol/L	165 (139 - 193)	176 (140 - 194)	0.948
sCrea_baseline,_ µmol/L	84 (77 - 106)	108 (81 - 141)	0.148
ΔsCrea*, µmol/L	35.2 (26.4 - 52.8)	8.8 ((- 8.8) - 8.8)	< 0.001
sCrea_max_, µmol	176 (132 - 282)	132 (106 - 167)	0.082
Variables are given as median and interquartile range; age is given as median (min -max). P-values were calculated using Wilcoxon rank-sum test (significance level α = 0.05); for categorical variables Chi-square and Fisher’s tests were used. AKI - acute kidney injury. No-AKI - no acute kidney injury. Euroscore - European System for Cardiac Operative Risk Evaluation. NGAL - neutrophil gelatinase-associated lipocalin. sCrea - serum creatinine. CKD - chronic kidney disease. *ΔsCrea was obtained as difference between serum creatinine concentration after and before surgery.			

Postoperative AKI occurred in 24 (0.35) of the 69 patients. Preoperative eGFR < 60 mL/min/1.73 m^2^ was present in 45 patients, 33 in No-AKI group and 12 in AKI patients.

**Figure 2:**
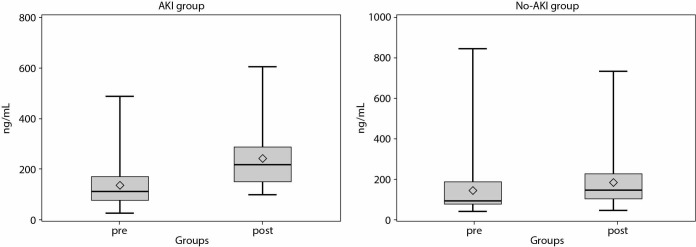
Plasma neutrophil gelatinase-associated lipocalin (NGAL) tests (ng/mL) expressed as mean ± standard deviation values at the time baseline (pre) and post cardiac surgery. The differences between NGAL pre and NGAL post become statistically significant only in the AKI group. AKI - acute kidney injury. No-AKI - no acute kidney injury.

**Figure 3:**
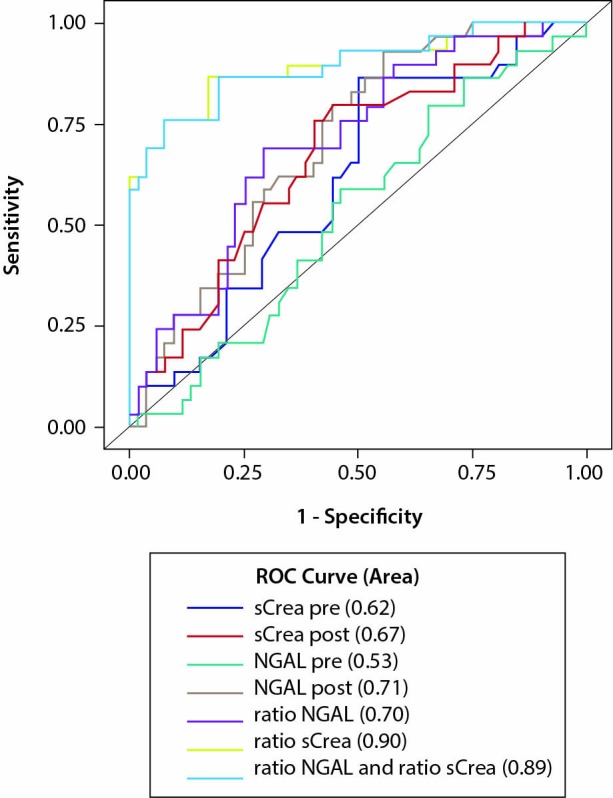
Receiver operating characteristic (ROC) curve analysis with comparison between the neutrophil gelatinase-associated lipocalin (NGAL) and serum creatinine (sCrea) curves. The better area under the curves (AUCs) were obtained using two NGAL and sCrea tests.

Pre-operative NGAL concentrations, expressed as median (50th percentile), 25th percentile, 75th percentile, were not different in patients with AKI compared with those without AKI. In both groups NGAL increased from baseline to after surgery reaching significantly higher concentrations only in AKI group (No-AKI: pre 145 ± 94 ng/mL, post 166 ± 135 ng/mL, P = 0.08; AKI: pre 125 ± 98 ng/mL, post 241 ± 133 ng/mL, P < 0.001) (Figure 2). We evaluated the ratio of postoperative (post-NGAL) to baseline (pre-NGAL): in No-AKI patients a ratio of 1.4 was found, while AKI subjects presented a ratio always above 1.5 (AKI1: 1.8, AKI2: 3.2, AKI3: 2.8). In particular, a ratio of 1.7 discriminates No-AKI patients from AKI patients with a sensitivity of 68% and a specificity of 72%. Receiver operating characteristic curve analysis of pre-operative and post-operative NGAL and pre and post-operative sCrea are shown in Figure 3. Post-operative NGAL showed AUC of 0.71, 95% CI (0.60 - 0.82) with a sensitivity of 76% and a specificity of 59% at the cut-off of 154 ng/mL. This NGAL concentration, to discriminate AKI or not AKI risk patients, showed a limitation with a poor specificity as well as described in literature, based on the comparison of a single determination to the cut-off (*16*-*24*). Instead, considering our methodology, we assessed two consecutive NGAL tests, with analysis of the increase of biomarker concentration, obtaining better results in the ROC curves analysis (P < 0.005) (Figure 3). Table 2 shows the association between NGAL values and the severity of AKI, (AKI 1, 2, 3) *vs* (AKI 2, 3) (P ≤ 0.001), showing a higher NGAL sensitivity and negative predictive value (NPV) with the serum creatinine combination.

**Table 2:** Neutrophil gelatinase-associated lipocalin diagnostic accuracy calculated by two consecutive tests and referred to AKI stage

	**Sensitivity** **(95% CI)**	**Specificity** **(95% CI)**	**NPV**
**Stage AKI 1, 2 and 3**			
ΔNGAL post ≥ 100	86% (76 - 92)	51% (40 - 62)	87%
*ΔNGAL post ≥ 100 and ΔsCr > 14%	79% (68 - 87)	83% (72 - 90)	88%
**Stage AKI 2 and 3**			
ΔNGAL post ≥ 100	93% (84 - 97)	43% (32 - 55)	97%
*ΔNGAL post ≥ 100 and ΔsCr > 14%	86% (76 - 92)	70% (59 - 80)	96%
AKI - acute kidney injury. NPV - negative predictive value. NGAL - neutrophil gelatinase-associated lipocalin. sCrea - serum creatinine. *Δ represents increase of biomarker concentration and was obtained as difference between biomarker concentration after and before surgery.			

Given the reference change value (RCV) of creatinine equal to 14%, evaluated through the analytical and biological variability, we divided patients into two groups, one with creatinine increase < 14% and the other with an increase of ≥ 14%. This approach of biomarker combination introduces the possibility to have an early confirmation of AKI prediction, at 10-18 h after surgery.

## Discussion

A reliable biomarker for predicting AKI after cardiac surgery would result in a more timely therapeutic intervention, limiting the associated morbidity. In our population of high-risk patients undergoing cardiac surgery we found a 35% incidence of AKI. Plasma NGAL concentrations measured soon after the arrival in ICU were associated with AKI, with a good sensitivity but with modest specificity. Much better diagnostic information was obtained combining two consecutive tests of NGAL immediately after surgery or combining the NGAL increase with the increase of serum creatinine, 10-18 hours after the end of surgical procedure.

Neutrophil gelatinase-associated lipocalin has been extensively investigated for the early recognition of tubular damage: in fact, it is up regulated after ischemic damage and may precede the increase in serum creatinine of 1-3 days. A recent meta-analysis suggested that NGAL measured within 6 h after the end of cardiac surgery might be a promising biomarker of AKI, although no definite agreement exists on the threshold to be used (*24*).

In agreement with the results found by other groups, we found comparable concentrations of NGAL in the preoperative samples, while in postoperative samples NGAL increased slightly in No-AKI patients and significantly in AKI patients, with higher concentrations in cases of more severe kidney impairment. However, some authors did not demonstrate this association (*16*). Friedrich *et al.* examined 81 patients undergoing cardiopulmonary bypass and found that, although NGAL increased after surgery, the peak value neither predict AKI nor the severity of the injury (*16*); in a similar population Perry *et al.* found that, although NGAL increases were associated with AKI, however, due to the low sensitivity (38.7%) its evaluation has limited utility (*17*). Different explanations may be considered for these different conclusions: the sample used (plasma or urine), the timing of sampling, the age at surgery, the presence of comorbidities.

A post-operative NGAL of 154 ng/mL was found as the best threshold: in fact all the 12 patients with AKI2 and AKI3, except one who presented a concentration of 149 ng/mL, had a higher concentration. This data are in line with what found in a study focused on patients with chronic kidney failure undergoing cardiac surgery: the authors analysed 166 patients and found that a value of NGAL of 155 ng/mL was the best threshold, with a sensitivity of 79% and a specificity of 58%, quite identical with our findings (*25*).

A more interesting finding is related to the use of the NGAL increases (ΔNGAL) between postoperative and preoperative NGAL. We found a significant difference between No-AKI and AKI groups. Patients who did not develop AKI showed a slight and not significant increase in NGAL, while patients with AKI showed a significant increase, with patients with a more severe kidney injury having the higher concentrations.

The delta evaluation could represent a solid method to detect changes of the biochemical biomarker showing a worthy diagnostic significance. Indeed, the utility of sequential measurements in patients with preoperative renal impairment has been recently suggested: an increase of NGAL with the second value at least of 100 ng/mL was proposed as possible and indicative tubular damage (*15*). We propose this value (determined on the post-surgery sample) and in fact, looking at our patients with severe AKI the mean delta was 115 ng/mL, with only two patients showing lower increases. However, one of them had already elevated NGAL before surgery, probably indicating the presence of a preoperative serious renal impairment with an eGFR value of 52 mL/min/1.73m^2^.

To further increase the overall performance we combined the results of NGAL and creatinine measurements. This approach results in an increase in both sensitivity and specificity, and might represent a good choice according to recent study too on the cardiac surgery-associated acute kidney injury (CSA-AKI) (*24*, *26*). However while NGAL values were available within few hours after the end of surgery, creatinine was determined using the RCV at 10-18 h after surgery. A possibility could be a two-step approach: patients with an increase of NGAL ≥ 100 ng/mL could be considered at high risk of AKI and may receive immediate treatment or more appropriately, patients with NGAL increase below 100 ng/mL, could be excluded for AKI risk. For all the other patients the evaluation can be completed on the first post-operative day with the determination of serum creatinine. Using this strategy we would reach a very high sensitivity and an acceptable specificity for the rule out of cardiac patients in ICU.

Our work has some limitations. First, it was conducted in a centre with patients at high-risk of AKI, undergoing elective cardiac surgery and the results obtained should be strictly referred at the clinical conditions adopted. However, the incidence of AKI is quite similar to what reported in developed country and therefore our population can be considered representative of the routine in cardiac surgery. Second, although patients with severe AKI represented the 35% of the surgical population, we still have to confirm our results on a larger number of patients. However, present data could be challenging to start a clinical trial or to plan NGAL measurements in an intensive clinical setting. Indeed, we have emphasized that the NGAL biomarker may be applied in the clinical practice, improving the diagnostic accuracy by the measure of two consecutive tests. We believe that, our study should be methodologically intended as a step forward for the AKI biomarkers underlying his diagnostic value even for the cardiovascular events associated to the kidney impairment (*23*-*28*). In conclusion the results obtained in this research demonstrate that plasma NGAL measured at baseline and early after cardiac surgery was able to predict the development of AKI after cardiac surgery, and that the combination with the changes in creatinine may be a useful tool to select patients at risk and to start early appropriate treatments.
